# UVC disinfects SARS-CoV-2 by induction of viral genome damage without apparent effects on viral morphology and proteins

**DOI:** 10.1038/s41598-021-93231-7

**Published:** 2021-07-05

**Authors:** Chieh-Wen Lo, Ryosuke Matsuura, Kazuki Iimura, Satoshi Wada, Atsushi Shinjo, Yoshimi Benno, Masaru Nakagawa, Masami Takei, Yoko Aida

**Affiliations:** 1 Laboratory of Global Infectious Diseases Control Science, Graduate School of Agricultural and Life Sciences, the University of Tokyo, 1-1-1 Yayoi, Bunkyo-ku, Tokyo, 113-8657 Japan; 2 Division of Hematology and Rheumatology, Department of Medicine, Nihon University School of Medicine, 30-1 Oyaguchi, Kami-Cho, Itabashi, Tokyo 173-8610 Japan; 3 Farmroid Co.,Ltd., 3-22-4 Funado, Itabashi-ku, Tokyo, 174-0041 Japan; 4 Photonics Control Technology Team, RIKEN Center for Advanced Photonics, 2-1 Hirosawa, Wako, Saitama 351-0198 Japan; 5 Benno Laboratory, Baton Zone Program, RIKEN Cluster for Science, Technology and Innovation Hub, 2-1 Hirosawa, Wako, Saitama 351-0198 Japan

**Keywords:** Microbiology, Optics and photonics

## Abstract

Severe acute respiratory syndrome coronavirus 2 (SARS-CoV-2) has been a pandemic threat worldwide and causes severe health and economic burdens. Contaminated environments, such as personal items and room surfaces, are considered to have virus transmission potential. Ultraviolet C (UVC) light has demonstrated germicidal ability and removes environmental contamination. UVC has inactivated SARS-CoV-2; however, the underlying mechanisms are not clear. It was confirmed here that UVC 253.7 nm, with a dose of 500 μW/cm^2^, completely inactivated SARS-CoV-2 in a time-dependent manner and reduced virus infectivity by 10^–4.9^-fold within 30 s. Immunoblotting analysis for viral spike and nucleocapsid proteins showed that UVC treatment did not damage viral proteins. The viral particle morphology remained intact even when the virus completely lost infectivity after UVC irradiation, as observed by transmission electronic microscopy. In contrast, UVC irradiation-induced genome damage was identified using the newly developed long reverse-transcription quantitative-polymerase chain reaction (RT-qPCR) assay, but not conventional RT-qPCR. The six developed long RT-PCR assays that covered the full-length viral genome clearly indicated a negative correlation between virus infectivity and UVC irradiation-induced genome damage (R^2^ ranging from 0.75 to 0.96). Altogether, these results provide evidence that UVC inactivates SARS-CoV-2 through the induction of viral genome damage.

## Introduction

The novel coronavirus, severe acute respiratory syndrome coronavirus 2 (SARS-CoV-2), causing coronavirus disease 2019 (COVID-19) was identified in the Hubei province of China in December 2019 before spreading worldwide and causing serious health and economic burdens^1^. More than 79 million people have been infected, with a 2.2% death rate, as of 27 December 2020 (https://is.gd/WQEhWz). SARS-CoV-2 is an enveloped, single-positive sense RNA virus belonging to the *Coronaviridae* family^2^. The genome of SARS-CoV-2 is approximately 30 kb, encoding the genes for four structural proteins, two large replicase polyproteins which will be proteolytic cleaved into 16 putative non-structural proteins for viral replication/ transcription, and other small proteins with unknown function^3–5^. The spike (S) structural protein of SARS-CoV-2 is stalked on the virus membrane and is responsible for viral entry by interacting with the host receptor, angiotensin converting enzyme II^6^. The envelope (E) protein of coronaviruses participates in virus assembly, budding, morphogenesis, and trafficking^7^. The membrane (M) protein of coronaviruses is the most abundant glycoprotein in the virion and acts as a central organizer of virus assembly^8^. The nucleocapsid (N) protein of coronaviruses packages and protects the viral genome by binding to it and forming the ribonucleocapsid^9^.

The rapid outbreak of SARS-CoV-2 is due to its high transmission ability. The basic reproductive rate (R_0_) for SARS-CoV-2 is estimated to be 2.5 (range 1.8–3.6), compared to 2.0 for SARS-CoV, 1.5 for the 2009 influenza pandemic, and 3.0 for the 1918 influenza pandemic^10^. There are several routes of SARS-CoV-2 transmission that have been proposed, including air-borne transmission, surface contamination, and fecal–oral transmission^11^. Consistently, viral RNA is detectable in a patient’s quarantine environment, including personal items, room surfaces, and toilets. Furthermore, infectious virus is detected from hallway air samples and windowsills, suggesting the risk of virus transmission from a contaminated environment^12,13^. The half-life of SARS-CoV-2 on different materials has been reported; in aerosol, it is approximately 1.1 h, for stainless steel, it is 5.6 h, and for plastic, it may be as long as 6.8 h^14^. These results strongly emphasize the importance of surface and environmental disinfection to reduce the risk of SARS-CoV-2 transmission.

Ultraviolet (UV) light is a potent disinfectant; based on wavelength, UV is classified as UVA (320–400 nm), UVB (280–320 nm), and UVC (200–280 nm)^15^. UV wavelength is negatively related to anti-microbial ability; therefore, UVC is the most powerful UV light among the three that has been used in clinical disinfection with efficient disinfection ability^16^. Several mechanisms of virus inactivation by UVC have been reported. For example, UVC causes viral protein oxidation, which is linked to the reduction of virus infectivity in feline calicivirus and bacterial phage MS2^17–19^. Second, UVC irradiation destroys murine norovirus 1 (MNV-1) viral capsid protein^20^. Third, UVC-induced virus protein-genome crosslinking is observed in poliovirus^21^. Fourth, UVC irradiation damages the genome of influenza virus^22^. However, UVC-induced damage varies in different virus types, as UVC damages the viral genome, but not viral proteins in adenovirus^23^. Consequently, the disinfection mechanism of UVC in different viruses requires testing.

Several findings^12–14^ highlight the importance of environmental contamination (air, liquid, and solid surfaces) and dissemination of SARS-CoV-2. UVC, which is the most powerful UV light, may be the most useful method to rapidly reduce SARS-CoV-2 viability in the environment. In fact, UVC has inactivated SARS-CoV-2 in different surfaces materials^24–26^. However, the mechanism of how UVC disinfects SARS-CoV-2 is unclear. Here, UVC mechanisms of SARS-CoV-2 inactivation were investigated by viral morphology analysis using transmission electronic microscopy (TEM), protein damage testing using immunoblotting, and viral genome integrity inspection using reverse-transcription quantitative-polymerase chain reaction (RT-qPCR) and long-RT PCR.

## Results

### Schematic of the UVC exposure system to inactivate SARS-CoV-2

**Figure 1:**
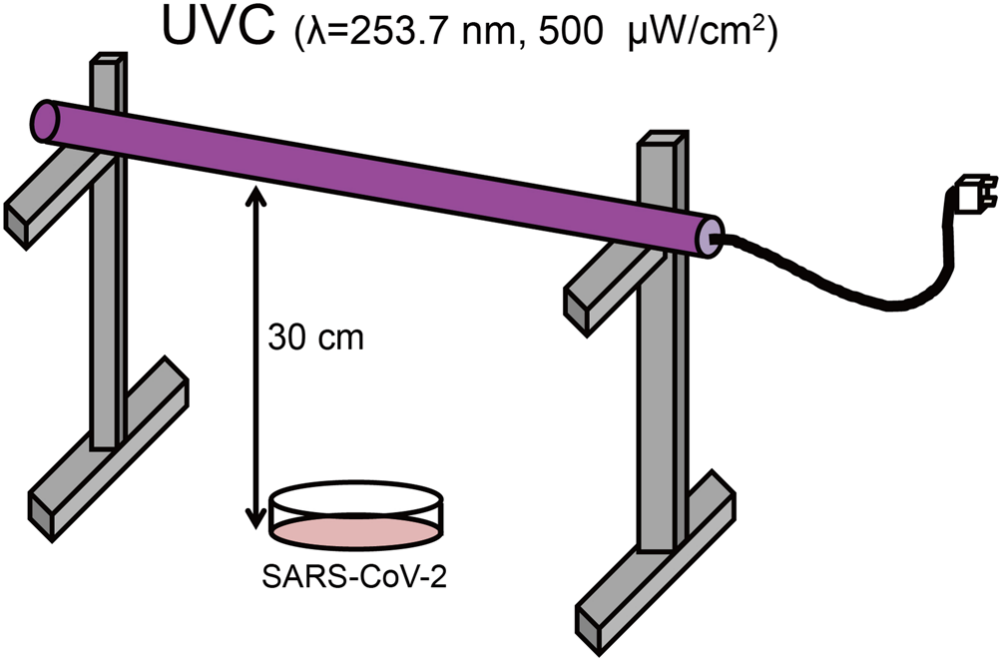
The ultraviolet C (UVC) light irradiation system. A UVC wavelength of 253.7 nm, 500 μW/cm^2^ is used. The length and diameter of the light tube are 295 ± 3 mm and 15.5 ± 0.5 mm, respectively. SARS-CoV-2 (5 mL) with a titer of 5 × 10^4^ 50% tissue culture infective dose (TCID_50_/mL) or (1 mL) with a titer of 1.78 × 10^6^ TCID_50_/mL are placed in a 10 cm^2^ dish, 30 cm below the UVC light tube, and are continuously and individually irradiated using a UVC lamp for 0, 5, 15 or 30 s.

Among various UVC light sources, a low pressure mercury lamp was used for inactivation of SAES-CoV-2, because it is more practical that has high average power with low cost. It generates the UV light at 253.7 nm. In previous works of UVC inactivation of corona viruses, dose of light were typically 2 mW/cm^2^^24,27^. Therefore, we start the intensity of light and exposure time as 500 μW/cm^2^ and 5 s. For UVC irradiation, a UVC light tube was set at a 30 cm height inside a safety cabinet. The virus was placed in a 10 cm^2^ dish beneath the UVC light tube (Fig. 1).

### UVC inactivated SARS-CoV-2 in a time-dependent manner

**Figure 2:**
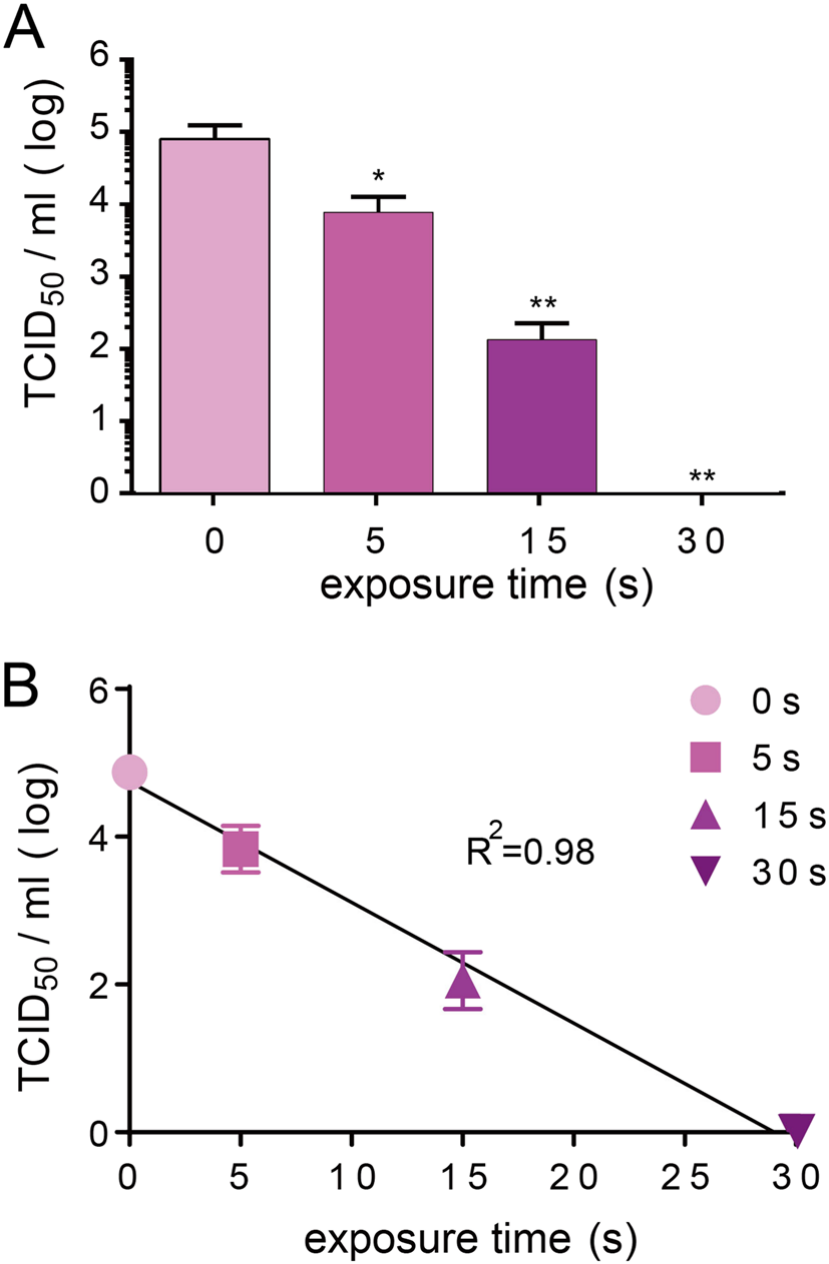
Effect of ultraviolet C (UVC) irradiation on severe acute respiratory syndrome coronavirus 2 (SARS-CoV-2) infectivity. (**A)** Change in 50% tissue culture infective dose (TCID_50_) of SARS-CoV-2 after UVC irradiation. SARS-CoV-2 (5 mL) with a titer of 5 × 10^4^ TCID_50_/mL was irradiated with a UVC light tube for 0, 5, 15 or 30 s and then was titrated using the TCID_50_ assay with Vero E6/TMPRSS2 cells. Assays are in triplicate and values represent the mean ± standard deviation (SD) of three replicates. Significance has been determined using one-way analysis of variance followed by Dunnett’s multiple comparisons test. The asterisk indicates a statistical difference (*p < 0.05; **p < 0.01). (**B)** Linear regression analysis to examine the correlation between UVC exposure time and SARS-CoV-2 infectivity.

To measure the SARS-CoV-2 inactivation efficiency of UVC, virus-containing medium was exposed to UVC for increasing periods of time from 5 to 30 s (Fig. 1). Virus titers after UVC irradiation were then quantified using a TCID_50_ assay with VeroE6/TMPRSS2 cells. As shown in Fig. 2A, UVC irradiation for 5, 15 and 30 s significantly (*p* < 0.05 and *p* < 0.01) decreases virus infectivity by a reduction of 1.0, 2.8, and 4.9 log10, respectively, and replication of SARS-CoV-2 completely ceases after 30 s. In addition, linear regression analysis indicated that UVC inactivated SARS-CoV-2 in a time-dependent manner and it was estimated that 29 s of UVC irradiation fully inhibited SARS-CoV-2 infectivity (R^2^ = 0.98) (Fig. 2B).

### UVC exposure had no effect on SARS-CoV-2 virion morphology

**Figure 3:**
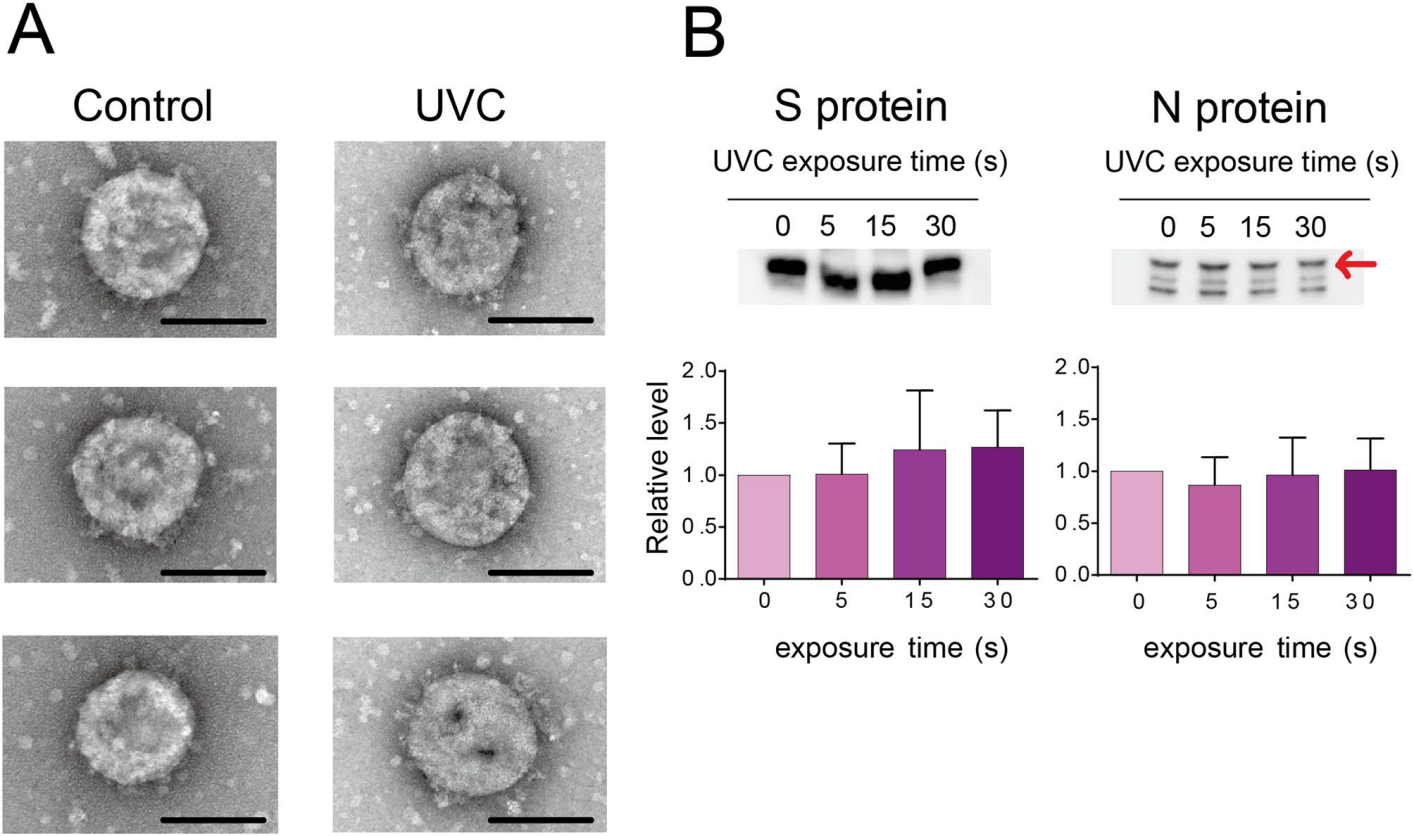
Effect of ultraviolet C (UVC) irradiation on viral morphology and viral proteins. (**A)** Representative viral particle images analyzed by transmission electron microscopy (TEM, three pictures for each group). SARS-CoV-2 (1 mL) with a titer of 1.78 × 10^6^ 50% tissue culture infective dose (TCID_50_/mL) was irradiated with a UVC light tube for 60 s and then analyzed by TEM (Scale bar of 100 nm). (**B)** Representative immunoblot images of viral spike (S) and nucleocapsid (N) proteins. SARS-CoV-2 (1 mL) with a titer of 1.78 × 10^6^ TCID_50_/mL was irradiated with a UVC light tube for increasing periods of time from 0 to 30 s. Equal volumes of irradiated virus suspension were then used for immunoblotting analysis using anti-SARS-CoV-2 spike monoclonal antibody (1A9) and anti-SARS-CoV-2 nucleocapsid monoclonal antibody (6H3) (upper panel). Positions of the S and N proteins are indicated. Full-length blots are shown in Supplementary Fig. S1. The intensity of both proteins is analyzed using CS Analyzer 4 software and quantitative results are shown in the bar diagram (lower panel). Data in the plot represent the mean ± standard deviation (SD) of three replicates. Significance has been determined using one-way analysis of variance followed by Dunnett’s multiple comparisons test.

As UVC had potential to induce viral protein damage, whether UVC destroyed virion structure was tested using TEM analysis (Fig. 3A). The SARS-CoV-2 particle is a round shape, approximately 100 nm in diameter and stalked with the spike protein on the surface. After 1 min of UVC irradiation, the UVC-treated virus particle also showed a similar structure, while the virus totally lost infectivity. These results suggested that UVC inactivated virus infectivity, but had no apparent effect on viral particle morphology.

### UVC did not degrade SARS-CoV-2 spike (S) and nucleocapsid (N) proteins

To further investigate the effect of UVC on viral proteins, immunoblotting was performed to analyze the integrity of S and N proteins after UVC irradiation (Fig. 3B). The 180 kDa and 55 kDa bands were identified as S and N proteins, respectively (Fig. 3B upper panel). The blot intensities of the control and UVC-treated group showed no significant differences for both proteins (Fig. 3B lower panel). These results suggested that protein degradation might not be the major reason for UVC-induced virus inactivation. Note that the measurement was based on two monoclonal antibodies, whether there were protein damages that occurred outside the epitope regions need further investigation.

### UVC damaged the SARS-CoV-2 genome

**Figure 4:**
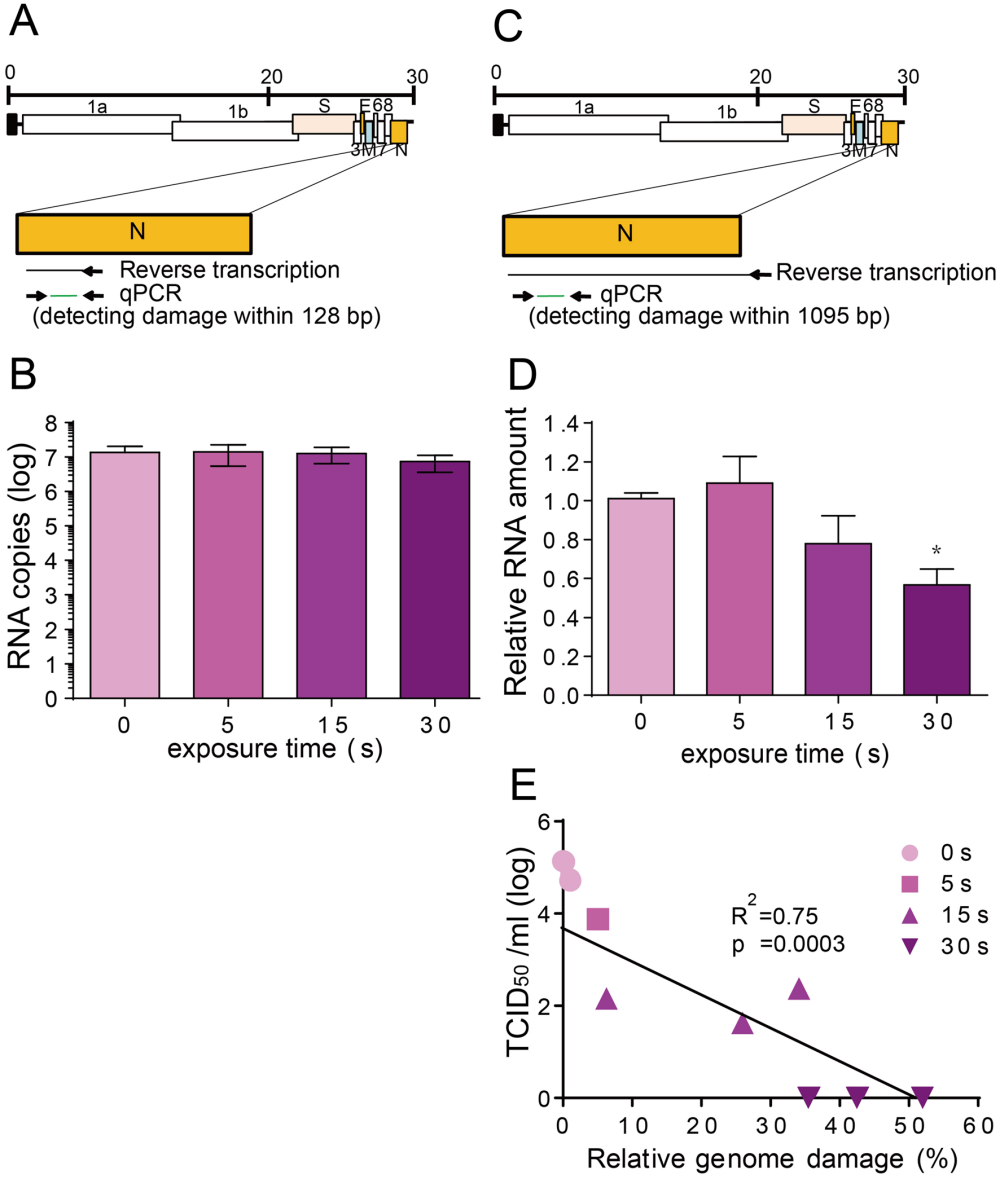
Reverse-transcription quantitative-polymerase chain reaction (RT-qPCR) to determine the effect of ultraviolet C (UVC) irradiation on the viral genome. (**A**) Schematic diagram of the conventional RT-qPCR RT-priming site and qPCR primer sites. (**B**) Quantitative results of conventional RT-qPCR. SARS-CoV-2 (5 mL) with a titer of 5 × 10^4^ 50% tissue culture infective dose (TCID_50_/mL) was irradiated with a UVC light tube for increasing periods of time from 0 to 30 s; the viral RNA was extracted and then viral RNA copy number was determined. (**C)** Schematic diagram of the long RT-qPCR RT-priming site and qPCR primer sites. (**D**) Quantitative results of long RT-qPCR. SARS-CoV-2 (5 mL) with a titer of 5 × 10^4^ TCID_50_/mL was irradiated with a UVC light tube for increasing periods of time from 0 to 30 s. The relative RNA fold change compared to 0 s irradiation is calculated. Data in the plot represent the mean ± standard deviation (SD) of three replicates. Significance has been determined using one-way analysis of variance followed by Dunnett’s multiple comparisons test. The asterisk indicates a statistical difference (*p < 0.05). (**E**) Linear regression analysis between relative viral genome damage and virus infectivity (derived from Fig. 2A) based on the long-RT-qPCR results. Relative genome damage is calculated as follows: (1-relative RNA fold change compared to control) × 100%. The data points that outside the axis limits are not shown in the graph.

To clarify the mechanism of SARS-CoV-2 inactivation by UVC, RT-qPCR was performed to detect the damage of the viral genome. First, conventional RT-qPCR was performed, which detected genome damage of 128 bp within the N gene (Fig. 4A). After UVC irradiation, amounts of viral RNA showed no difference compared to the control group (Fig. 4B). These results are inconsistent with previous reports^28^. However, this observation might be due to the detection limitation of conventional RT-qPCR, which only detects a small region of the viral genome. Therefore, next, viral genome damage was tested using a long RT-qPCR that covered 1095 bp of the N gene region (Fig. 4C). Amounts of viral RNA decreased with UVC irradiation in a time-dependent manner, and 30 s UVC irradiation significantly decreased SARS-CoV-2 RNA levels to 56.6% (Fig. 4D). Linear regression analysis (Fig. 4E) of viral infectivity (derived from Fig. 2A) with genome damage indicated a negative correlation (R^2^ = 0.75). Although the long RT-qPCR only targeted 1095 bp, 3.8% of the full-length genome, 43.4% of virus was detected with RNA damage, suggesting that potentially almost all virions had RNA genome damage after 30 s of UVC irradiation.

**Figure 5:**
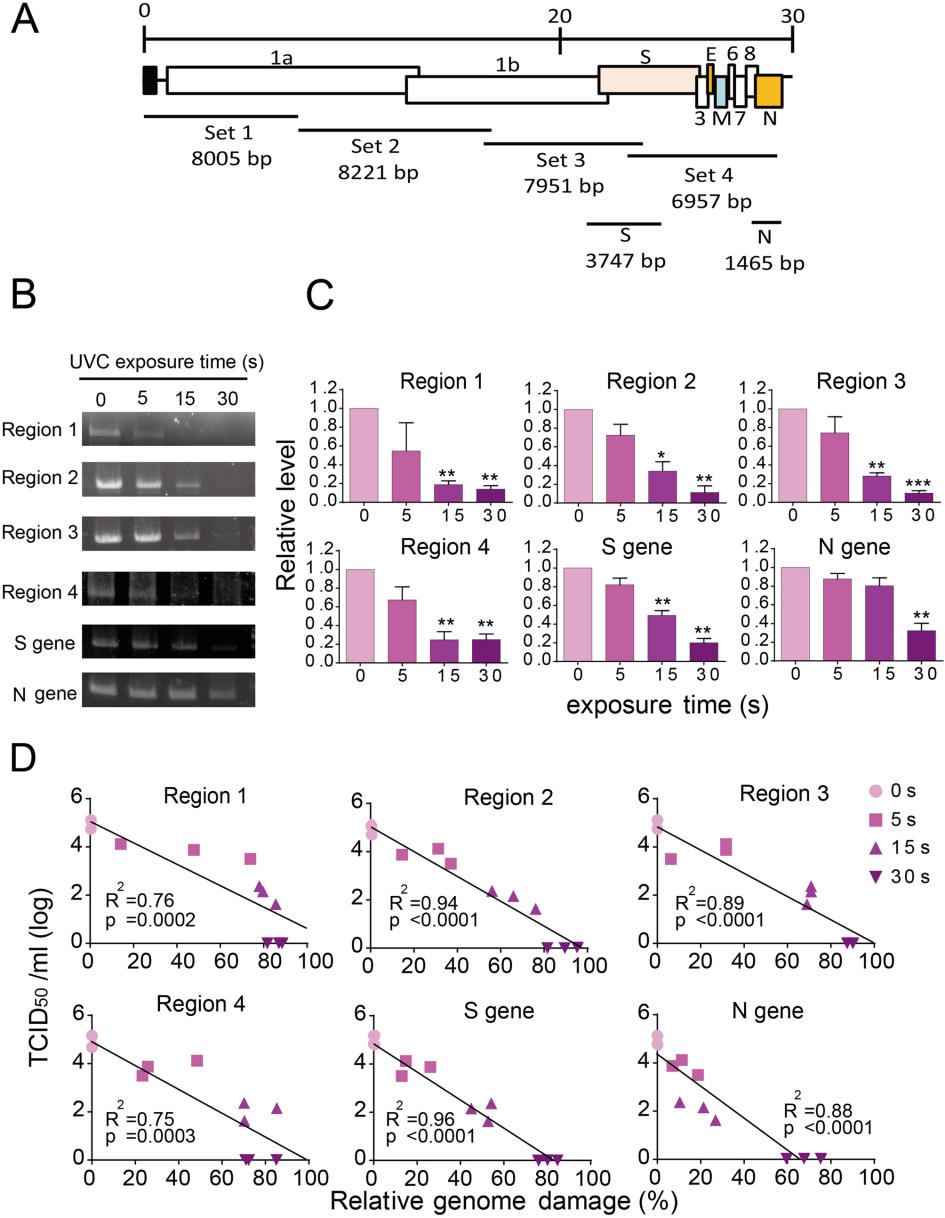
Long reverse-transcription polymerase chain reaction (RT-PCR) to determine the effect of ultraviolet C (UVC) irradiation on the viral genome. (**A**) Schematic diagram of the six pairs of long RT-PCR primer sets. (**B**) Representative PCR products are visualized by agarose gel electrophoresis. Full-length gels are shown in Supplementary Fig. S2. SARS-CoV-2 (5 mL) with a titer of 5 × 10^4^ 50% tissue culture infective dose (TCID_50_/mL) was irradiated with a UVC light tube for increasing periods of time from 0 to 30 s. Viral RNA was reverse transcribed with the reverse primer for each region and then amplified with the corresponding primer sets. The amplified PCR products were used for 0.8% agarose gel electrophoresis and detected by ethidium bromide staining. (**C**) Band intensity is analyzed using CS Analyzer 4 software and quantitative results are shown in the bar diagram. Data in the plot represent the mean ± standard deviation (SD) of three replicates. Significance has been determined using one-way analysis of variance followed by Dunnett’s multiple comparisons test. The asterisk indicates a statistical difference (*p < 0.05; **p < 0.01; ***p < 0.001). (**D**) Linear regression analysis between relative viral genome damage and virus infectivity (derived from Fig. 2A). Relative genome damage is calculated as follows: (1-relative PCR fold change compared to control) × 100%.

Next, to further confirm that UVC caused SARS-CoV-2 RNA damage, four primer sets that covered the full-length viral genome were used for long RT-PCR analysis (target size for region 1: 8005 bp, region 2: 8221 bp, region 3: 7951 bp, and region 4: 6957 bp), together with the S gene (target size was 3747 bp) and the N gene (target size was 1465 bp) (Fig. 5A). As shown in Fig. 5B, all four regions and the S gene showed RNA damage after UVC irradiation in a time-dependent manner: Band signals in agarose gels were barely detectable after 30 s of UVC irradiation, suggesting that almost the entire virus genome contained RNA damage. Note that for the N gene region, signals were detectable after 30 s of UVC irradiation, which totally inactivated virus infectivity (Fig. 5B). This might be because the detection range of the N gene was relatively short and not all the UVC-irradiated virus contained genome damage within this region. In the quantitative analysis, band intensities of the control and UVC-treated group after 30 s of UVC irradiation consistently showed significant differences for all six regions; however, there was no significant difference in the N gene band intensity after 15 s of UVC irradiation (Fig. 5C). Moreover, by linear regression analysis (Fig. 5D), the RNA damage caused by UVC irradiation was strongly correlated with virus infectivity (derived from Fig. 2A) in all regions tested: region 1 (R^2^ = 0.76), region 2 (R^2^ = 0.94), region 3 (R^2^ = 0.89), region 4 (R^2^ = 0.75), S gene (R^2^ = 0.96) and the N gene (R^2^ = 0.88). Taken together, these results indicated that genome damage was the main mechanism of UVC-induced SARS-CoV-2 inactivation.

## Discussion

Implementation of environmental cleaning and disinfection by irradiation with UVC adequately removes microbial contamination from the environment. Indeed, UVC has recently inactivated SARS-CoV-2 and therefore may be applied in healthcare facilities for environmental disinfection^29^. However, the mechanism of action of how UVC inactivates SARS-CoV-2 is unclear. In this study, UVC efficiently disinfected SARS-CoV-2, mainly through viral genome damage. By transmission electronic microscopy observation and immunoblotting analysis with two monoclonal antibodies, no apparent effects on viral morphology and viral proteins were found. However, by the limitation of our experiment, we could not conclude that UVC does not damage proteins of SARS-CoV-2. In conclusion, our results support the idea that UVC is an effective countermeasure for SARS-CoV-2 disinfection in high-risk places, such as hospitals and airports.

There are two possible hypotheses regarding the mechanism of virus disinfection by UVC, either viral genome or viral protein damage. For damage to the viral genome, the UVC wavelength ranges from 200 to 280 nm, which is close to the absorption wavelength of nucleic acids, and thus may induce damage to the viral genome^30^. Our results demonstrated that genome damage was the main mechanism of UVC-induced SARS-CoV-2 inactivation. At the protein level, UVC treatment causes photo-damage to proteins through disulfide photolysis and oxygen radical oxidation^31^. Additionally, a report shows that UVC irradiation decreases MNV-1 capsid protein amounts, as measured by Coomassie staining^20^. However, by testing the effect of UVC on viral morphology and levels of SARS-CoV-2 viral spike/nucleocapsid proteins, no obvious protein damage was observed in this study. Indeed, UV-inactivated SARS-CoV vaccine has been shown the ability eliciting systemic humoral immunity against viral spike and nucleocapsid proteins in mice^32^, supporting the possibility that SARS-CoV-2 viral morphology and proteins are not under severe damage after UV treatment. One possible reason to explain the discrepancy is that viral proteins between different viruses might have different UVC susceptibilities, as adenoviral proteins have different susceptibilities to UVC^33^. In fact, UVC treatment of Tulane virus damages the viral capsid binding protein, whereas protein damage is not observed in porcine rotavirus under the same experimental condition^34^, supporting the idea that UVC inactivation mechanisms may vary across different viruses. A second reason is that the UVC wavelength used in each study might be different and therefore lead to inconsistent results of viral protein damage. Compared to UVC 254 nm, which is used in this research, UVC 220 nm more efficiently affects viral protein integrity in adenovirus^33^. A third possible reason might due to differences in the UVC fluency used in each publication. For example, MNV-1 protein is destroyed by UVC after 10 min of 8 W/cm^2^ irradiation, which is much higher than the current study (30 s of 500 μW/ cm^2^)^20^. A fourth reason may be oxidation of viral proteins during irradiation with UV 254 nm. For example, several viruses, including MS2, astrovirus, and norovirus are inactivated via oxidative damage of viral proteins^18,19^. Finally, in our experimental setting, we applied two monoclonal antibodies for testing protein damage; however, we could not rule out the possibility of existence of potential protein damage that occurred outside the antibodies recognition regions. Additional experiments, for example, detecting protein damage with polyclonal antibodies, mass spectrometry or NMR may provide substantial results for confirmation. In addition, whether UVC caused SARS-CoV-2 protein oxidation leading to a reduction in infectivity requires further investigation.

PCR-based methods e.g., qPCR is widely used for virus detection and infectivity measurements^35,36^. However, we and others have noted that UVC-induced viral genome damage is not detectable using conventional RT-qPCR, which is used in the clinic, even when the virus completely loses infectivity^28^. This inconsistency might lead to misinterpretation of environmental virus infectivity measurements after UVC disinfection. The reason for the detection failure of viral genome damage by conventional qPCR is due to its short range (< 200 bp); the damage is not detectable if it does not occur within this region. Therefore, analysis of longer regions of virus genome may more accurately inspect genome integrity. A similar phenomenon has been reported in the detection of UVC-induced damage in porcine parvovirus that tested viral genome damage using RT-PCR; the data shows that targeting a genome size of 2000 bp compared to 300 bp more accurately reflects virus infectivity^37^. In the current study, one long RT-qPCR (1095 bp) and six long RT-PCR assays (targeting regions ranging from 1465 ~ 8221 bp) were designed and applied to inspect viral genome damage that potentially includes strand break and pyrimidine dimer formation. Consistently, in the six long RT-PCR assays, regions 1 ~ 4, which target approximately 8000 bp, with 565–684 potential thymidine dimer sites, accurately reflected virus infectivity, as the PCR signal was not detectable in the group with 30 s UVC irradiation, where the virus was completely inactivated. In contrast, slight bands were observed in the S gene amplification products (3747 bp, with 332 potential thymidine dimer sites) and a clear band was observed in the N gene (1465 bp, with 70 potential thymidine dimer sites) amplification products after 30 s irradiation, though the virus had totally lost infectivity. These results emphasize the importance of the PCR targeting length for genome damage analysis, even though the long RT-qPCR and six long RT-PCR assays correctly reflected UVC-induced genome damage with virus infectivity (R^2^ ranging from 0.75 to 0.96). Therefore, the long RT-qPCR and six long RT-PCR assays that were developed might contribute to rapid and accurate infectivity inspection of post UVC-irradiated environments.

There are various types of light-based technology for virus inactivation. Among all three types of UV, UVC 253.7 nm has the strongest antimicrobial activity^38^. However, care should be taken during application, as exposure to these lamps is associated with health risks involving damage to the eyes and skin^39^. UVC (222 nm) has recently shown potential for SARS-CoV-2 disinfection^27,28^. Interestingly, UVC 222 did not harm skin when used on a mouse model, and therefore, has the capacity to be applied on external areas^40^. However, the safety determination of UVC 222 on humans requires further investigation. UVA, and UVB, which are present in natural sunlight with a lower risk to the human body, also inactivate SARS-CoV-2, although they have relatively low efficiency compared to UVC^24,41^. These results provide an alternative for UV-based disinfection, as UVC application should be avoided in live animals due to safety concerns. Blue light (380–500 nm) is less harmful to the human body compared to UV and exhibits germicidal activities, especially within the wavelength 400–500 nm^42^. LED blue light (413 nm) irradiation inactivating SARS-CoV-2 is documented^43^. The potential antiviral mechanism is via the generation of reactive oxygen species and thus, induces damage to proteins, lipids, and nucleic acids in the presence of photosensitizers^44^. Red light (600–700 nm) has lower risk for humans, but shows disinfection ability for Middle East Respiratory Syndrome-CoV and Ebola virus when combined with the use of methylene blue, which is widely used in plasma transfusions for pathogen reduction^45,46^. However, overall, red light-based inactivation is less efficient compared to UVC and non-enveloped viruses are less sensitive than enveloped viruses^45,47^. Taken together, all of the light-based technologies, with the different characteristics mentioned above, could be applied to virus inactivation. Based on the application purpose, the advantages and drawbacks of these tools should be evaluated. Thus, light-based technologies are a powerful and chemical-free agent that broadly disinfect various types of viruses. Therefore, these tools could potentially save people from the risk of infectious diseases. Furthermore, these technologies are also ideal for drug-resistant viruses, emerging viruses, such as Ebola virus, SARS-CoV, or other new infectious diseases in the future.

## Materials and methods

### Cells and virus

Vero E6/TMPRSS2 cells were obtained from the Japanese Collection of Research Bioresources (JCRB) Cell Bank in Japan (JCRB no. JCRB1819) and maintained in Dulbecco's modified Eagle medium (Thermo Fisher Scientific, Waltham, MA, USA) supplemented with 10% fetal bovine sera (FBS) (Sigma-Aldrich, St. Louis, MO, USA), 1% penicillin/streptomycin/glutamine (PSG), 2% G418 and cultured at 37 °C with 5% CO_2_. SARS-CoV-2 (SARS-CoV-2/JPN/TY/WK-521 strain) was a kind gift from the National Institute of Infectious Diseases of Japan^48^. For SARS-CoV-2 virus infection in VeroE6/TMPRSS2 cells, infection medium (Minimum Essential Medium Eagle (MEM, Thermo Fisher Scientific) containing 2% FBS and 1% PSG) was used. SARS-CoV-2 was propagated at a multiplicity of infection (MOI) = 10^–5^ for 3 days in Vero E6/TMPRSS2 cells. Viruses were titrated using 50% tissue culture infective dose (TCID_50_) assays. For the TCID_50_ assay, in brief, Vero E6/TMPRSS2 cells in a 96 well plate (2 × 10^4^ cells per well) were infected with 100 μL of tenfold serially diluted virus-containing infection medium (each dilution had eight replicates) and were incubated at 37 °C for 3 days. Following incubation, viral infection in each well was determined using the virus-induced cell cytopathic effect (CPE). Viral titers were calculated using the Reed-Muench method^49^.

### Inactivation of SARS-CoV-2 by UVC irradiation

A UVC light tube with a wavelength of 253.7 λ, 500 μW/cm^2^ (Bactericidal lamp GL16KSH; SANKYO DENKI Co., Ltd., Kanagawa, Japan), was set at a 30 cm height above the virus for the inactivation experiments (Fig. 1). The environmental temperature was controlled at 24 °C with 55% humidity. SARS-CoV-2 (5 mL) with a titer of 5 × 10^4^ TCID_50_/mL was placed in 10 cm dishes for UVC irradiation for 5, 15, or 30 s. The post-UVC irradiated virus was then titrated using the TCID_50_ assay.

### Virus morphology observation by TEM

SARS-CoV-2 (1 mL) with a titer of 1.78 × 10^6^ TCID_50_/mL was irradiated with a UVC light tube for 60 s. The post-irradiated virus (100 μL) was mixed with 2.5% glutaraldehyde (1 mL) for TEM negative staining. For TEM sample preparation, a droplet of the virus mixture was placed on a carbon-film grid for 10 s. After the grid was partially dried, a droplet of staining solution, 2% uranyl acetate, was added for a 10 s incubation. The excess liquid was wiped off with filter paper, and the grid was dried at room temperature before imagining using a HITACHI H-7600 electron microscope (Hitachi Global Life Solutions, Inc. Tokyo, Japan) at 100 kV.

### Viral protein damage detection by immunoblotting

SARS-CoV-2 (1 mL) with a titer of 1.78 × 10^6^ TCID_50_/mL was irradiated with a UVC light for 5, 15 or 30 s. A volume of 20 µL of virus-containing medium was mixed with 5 µL sample buffer (0.15 M Tris–HCl, 10% sodium dodecyl sulfate (SDS), 30% glycerol, and 0.5% bromophenol blue) and heated at 100 °C for 5 min. The 15 µL denatured virus solution was loaded onto a 10% (for spike protein detection) or 12% (for nucleocapsid protein detection) SDS–polyacrylamide gel and electrophoresed in running buffer containing 0.3% Tris, 0.1% SDS, and 1.44% glycine. The proteins were then transferred onto a polyvinylidene difluoride membrane (Millipore, Billerica, MA, USA) using a Trans-Blot Turbo apparatus (Bio-Rad, Hercules, CA, USA) and incubated with anti-SARS-CoV-2 spike monoclonal antibody (Mab) (1A9) (1:1000; Genetex, Irvine, CA, USA), anti-SARS-CoV-2 nucleocapsid Mab (6H3) (1:2000; Genetex) at 4 °C overnight. After washing, the membranes were incubated with horseradish peroxidase-conjugated AffiniPure goat anti-mouse IgG (1:2000; Jackson ImmunoResearch, West Grove, PA, USA) at room temperature for 1 h. Signals were visualized after treating the membrane with SuperSignal™ West Pico PLUS Chemiluminescent Substrate (Thermo Fisher Scientific). Images were acquired using a WSE-6100 LuminoGraph I (Atto Corporation, Tokyo, Japan). Densities of bands were analyzed using CS Analyzer ver.4 (Atto Corporation) (https://www.atto.co.jp/eng/products/geldocumentation/Image-analysis-software/Image-Analysis-Software2).

### RT-qPCR

SARS-CoV-2 (5 mL) with a titer of 5 × 10^4^ TCID_50_/mL was irradiated using a UVC light for 5, 15 or 30 s. Viral RNA was then extracted using a QIAamp Viral RNA Mini Kit (Qiagen, Hilden, Germany), following the manual instructions. For conventional RT-qPCR, a volume of 5 µL RNA was used for a one-step RT-qPCR reaction using a QuantiTect Probe RT-PCR Kit (Qiagen) with 600 nM forward primer (CACATTGGCACCCGCAATC), 800 nM reverse primer (GAGGAACGAGAAGAGGCTTG), 200 nM Taqman probe (FAM-ACTTCCTCAAGGAACAACATTGCCA-QSY), and an Applied Biosystems 7500 Fast Real-Time PCR system (Thermo Fisher Scientific), as reported previously^50^. For long RT-qPCR, a 5 µL volume of RNA was first used for reverse transcription with SARS-CoV-2 4R primer (CTCTTCCATATAGGCAGCTCT) using the SuperScript™ III First-Strand Synthesis System (Invitrogen, Foster, CA, USA), following the manual instructions. A total of 1.25 µL cDNA was used for the qPCR analysis, which was identical to the conventional RT-qPCR procedure above. Samples were evaluated in triplicate and data analysis was performed using the comparative CT method (∆∆CT).

### Long RT-PCR

SARS-CoV-2 (5 mL) with a titer of 5 × 10^4^ TCID_50_/mL was irradiated using UVC for 5, 15 or 30 s. Viral RNA was then extracted using a QIAamp Viral RNA Mini Kit (Qiagen), following the manual instructions. Reverse transcription was performed using the SuperScript™ III First-Strand Synthesis System (Invitrogen) and was followed by PCR amplification using PrimeSTAR GXL with a gene-specific primer. For region 1, RT was conducted using the SARS-CoV-2 reverse primer 1R2 (GAATCAACAAACCCTTGCCGA). The generated cDNA was then amplified with the SARS-CoV-2 primers 1F2 (GCTTACGGTTTCGTCCGTGT) and 1R2 using the following thermal cycling program: 98 °C for 1 min, followed by 30 cycles of 98 °C for 10 s, 57 °C for 15 s and 68 °C for 8 min. For region 2, RT was conducted using the SARS-CoV-2 reverse primer 2R (GACATCAGCATACTCCTGATTA). The generated cDNA was then amplified with the SARS-CoV-2 primers 2F (GACAACCTGAGAGCTAATAACAC) and 2R using the following thermal cycling program: 98 °C for 1 min, followed by 30 cycles of 98 °C for 10 s, 55 °C for 15 s and 68 °C for 8 min. For region 3, RT was conducted using the SARS-CoV-2 reverse primer 3R (TGCTGCATTCAGTTGAATCAC). The generated cDNA was then amplified with the SARS-CoV-2 primers 3F (GGACCTCATGAATTTTGCTCT) and 3R using the following thermal cycling program: 98 °C for 1 min, followed by 30 cycles of 98 °C for 10 s, 55 °C for 15 s and 68 °C for 8 min. For region 4, RT was conducted using the SARS-CoV-2 reverse primer 4R3 (GCCTCGGTGAAAATGTGGTG). The generated cDNA was then amplified with the SARS-CoV-2 primers 4F5 (TCAGACAAATCGCTCCAGGG) and 4R3 using the following thermal cycling program: 98 °C for 1 min, followed by 30 cycles of 98 °C for 10 s, 57 °C for 15 s and 68 °C for 7 min. For the S gene region, RT was conducted using the S gene reverse primer R2 (GACTCCTTTGAGCACTGGCT). The generated cDNA was then amplified with the S gene primers F2 (ACCAGAACTCAATTACCCCCTG) and R2 using the following thermal cycling program: 98 °C for 1 min, followed by 30 cycles of 98 °C for 10 s, 57 °C for 15 s and 68 °C for 4 min. For the N gene region, RT was conducted using the SARS-CoV-2 reverse primer 4R3. The generated cDNA was then amplified with the N gene primers F (ATGTCTGATAATGGACCCCA) and R (TTAGGCCTGAGTTGAGTCAG) using the following thermal cycling program: 98 °C for 1 min, followed by 30 cycles of 98 °C for 10 s, 57 °C for 15 s and 68 °C for 1.5 min. A volume of 5 μL of the amplified PCR products was then used for 0.8% agarose gel electrophoresis and bands were detected by ethidium bromide staining. The band intensities were quantitated using a WSE-6100 LuminoGraph I (Atto Corporation) and were analyzed using CS Analyzer ver.4 (Atto Corporation).

### Statistical analysis

All data are expressed as the mean ± standard deviation based on at least three independent experiments. Data were analyzed using analysis of variance with Dunnett’s multiple comparison test in Prism (GraphPad, San Diego, CA, USA). *p* values smaller than 0.05 were considered significant. Linear regression analysis of virus infectivity with UVC irradiation time, and virus infectivity with virus genome damage were analyzed using Prism. Virus genome damage was calculated as follows: (1-relative PCR fold change compared to control) × 100%.

## Supplementary Information

Supplementary Information.

## Data Availability

The datasets that support the finding of this study are available from the corresponding author upon reasonable request.

## References

[1] Wu F, Zhao S, Yu B, Chen Y-M, Wang W, Song Z-G, Hu Y, Tao Z-W, Tian J-H, Pei Y-Y (2020). A new coronavirus associated with human respiratory disease in China. Nature.

[2] Pal M, Berhanu G, Desalegn C, Kandi V (2020). Severe acute respiratory syndrome coronavirus-2 (SARS-CoV-2): An update. Cureus.

[3] Lu R, Zhao X, Li J, Niu P, Yang B, Wu H, Wang W, Song H, Huang B, Zhu N (2020). Genomic characterisation and epidemiology of 2019 novel coronavirus: implications for virus origins and receptor binding. The Lancet.

[4] Naqvi AAT, Fatima K, Mohammad T, Fatima U, Singh IK, Singh A, Atif SM, Hariprasad G, Hasan GM, Hassan MI (2020). Insights into SARS-CoV-2 genome, structure, evolution, pathogenesis and therapies: Structural genomics approach. Biochim. Biophys. Acta.

[5] Chan JF-W, Kok K-H, Zhu Z, Chu H, To KK-W, Yuan S, Yuen K-Y (2020). Genomic characterization of the 2019 novel human-pathogenic coronavirus isolated from a patient with atypical pneumonia after visiting Wuhan. Emerg. Microbes Infect..

[6] Hoffmann M, Kleine-Weber H, Schroeder S, Krüger N, Herrler T, Erichsen S, Schiergens TS, Herrler G, Wu N-H, Nitsche A (2020). SARS-CoV-2 Cell entry depends on ACE2 and TMPRSS2 and is blocked by a clinically proven protease inhibitor. Cell.

[7] Schoeman D, Fielding BC (2019). Coronavirus envelope protein: Current knowledge. Virol. J..

[8] Astuti I (2020). Severe acute respiratory syndrome coronavirus 2 (SARS-CoV-2): An overview of viral structure and host response. Diabetes Metab. Syndr..

[9] Zeng W, Liu G, Ma H, Zhao D, Yang Y, Liu M, Mohammed A, Zhao C, Yang Y, Xie J (2020). Biochemical characterization of SARS-CoV-2 nucleocapsid protein. Biochem. Biophys. Res. Commun..

[10] Petersen E, Koopmans M, Go U, Hamer DH, Petrosillo N, Castelli F, Storgaard M, Al Khalili S, Simonsen L (2020). Comparing SARS-CoV-2 with SARS-CoV and influenza pandemics. Lancet Infect. Dis..

[11] Mukhra R, Krishan K, Kanchan T (2020). Possible modes of transmission of Novel coronavirus SARS-CoV-2: A review. Acta Bio Med. Atenei Parmensis.

[12] Santarpia JL, Rivera DN, Herrera VL, Morwitzer MJ, Creager HM, Santarpia GW, Crown KK, Brett-Major DM, Schnaubelt ER, Broadhurst MJ (2020). Aerosol and surface contamination of SARS-CoV-2 observed in quarantine and isolation care. Sci. Rep..

[13] Chia PY, Coleman KK, Tan YK, Xiang Ong SW, Gum M, Lau SK, Sutjipto S, Lee PH, Son TT, Young BE (2020). Detection of air and surface contamination by severe acute respiratory syndrome coronavirus 2 (SARS-CoV-2) in hospital rooms of infected patients. medRxiv.

[14] van Doremalen N, Bushmaker T, Morris DH, Holbrook MG, Gamble A, Williamson BN, Tamin A, Harcourt JL, Thornburg NJ, Gerber SI (2020). Aerosol and surface stability of SARS-CoV-2 as compared with SARS-CoV-1. N. Engl. J. Med..

[15] Guerrero-Beltr JA, Barbosa GV (2004). Advantages and limitations on processing foods by UV light. Food Sci. Technol. Int..

[16] Casini B, Tuvo B, Cristina ML, Spagnolo AM, Totaro M, Baggiani A, Privitera GP (2019). Evaluation of an ultraviolet C (UVC) light-emitting device for disinfection of high touch surfaces in hospital critical areas. Int. J. Environ. Res. Public Health.

[17] Tanaka T, Nogariya O, Shionoiri N, Maeda Y, Arakaki A (2018). Integrated molecular analysis of the inactivation of a non-enveloped virus, feline calicivirus, by UV-C radiation. J. Biosci. Bioeng..

[18] Sano D, Pintó RM, Omura T, Bosch A (2010). Detection of oxidative damages on viral capsid protein for evaluating structural integrity and infectivity of human norovirus. Environ. Sci. Technol..

[19] Rule Wigginton K, Menin L, Montoya JP, Kohn T (2010). Oxidation of virus proteins during UV254 and singlet oxygen mediated inactivation. Environ. Sci. Technol..

[20] Park D, Shahbaz HM, Kim S-H, Lee M, Lee W, Oh J-W, Lee D-U, Park J (2016). Inactivation efficiency and mechanism of UV-TiO2 photocatalysis against murine norovirus using a solidified agar matrix. Int. J. Food Microbiol..

[21] Wetz K, Habermehl K-O (1982). Specific cross-linking of capsid proteins to virus RNA by ultraviolet irradiation of poliovirus. J. Gen. Virol..

[22] Nakaya Y, Fukuda T, Ashiba H, Yasuura M, Fujimaki M (2020). Quick assessment of influenza a virus infectivity with a long-range reverse-transcription quantitative polymerase chain reaction assay. BMC Infect. Dis..

[23] Bosshard F, Armand F, Hamelin R, Kohn T (2013). Mechanisms of human adenovirus inactivation by sunlight and UVC light as examined by quantitative PCR and quantitative proteomics. Appl. Environ. Microbiol..

[24] Heilingloh CS, Aufderhorst UW, Schipper L, Dittmer U, Witzke O, Yang D, Zheng X, Sutter K, Trilling M, Alt M (2020). Susceptibility of SARS-CoV-2 to UV irradiation. Am. J. Infect. Control.

[25] Sabino CP, Sellera FP, Sales-Medina DF, Machado RRG, Durigon EL, Freitas-Junior LH, Ribeiro MS (2020). UV-C (254 nm) lethal doses for SARS-CoV-2. Photodiagn. Photodyn. Ther..

[26] Gidari A, Sabbatini S, Bastianelli S, Pierucci S, Busti C, Bartolini D, Stabile AM, Monari C, Galli F, Rende M (2021). SARS-CoV-2 survival on surfaces and the effect of UV-C light. Viruses.

[27] Buonanno M, Welch D, Shuryak I, Brenner DJ (2020). Far-UVC light (222 nm) efficiently and safely inactivates airborne human coronaviruses. Sci. Rep..

[28] Kitagawa H, Nomura T, Nazmul T, Omori K, Shigemoto N, Sakaguchi T, Ohge H (2020). Effectiveness of 222-nm ultraviolet light on disinfecting SARS-CoV-2 surface contamination. Am. J. Infect. Control.

[29] Gilbert RM, Donzanti MJ, Minahan DJ, Shirazi J, Hatem CL, Hayward-Piatkovskyi B, Dang AM, Nelson KM, Bothi KL, Gleghorn JP (2020). Mask reuse in the COVID-19 pandemic: Creating an inexpensive and scalable ultraviolet system for filtering facepiece respirator decontamination. Glob. Health.

[30] Beck SE, Rodriguez RA, Linden KG, Hargy TM, Larason TC, Wright HB (2014). Wavelength dependent UV inactivation and DNA damage of adenovirus as measured by cell culture infectivity and long range quantitative PCR. Environ. Sci. Technol..

[31] Chan HL, Gaffney PR, Waterfield MD, Anderle H, Peter Matthiessen H, Schwarz HP, Turecek PL, Timms JF (2006). Proteomic analysis of UVC irradiation-induced damage of plasma proteins: Serum amyloid P component as a major target of photolysis. FEBS Lett..

[32] Takasuka N, Fujii H, Takahashi Y, Kasai M, Morikawa S, Itamura S, Ishii K, Sakaguchi M, Ohnishi K, Ohshima M (2004). A subcutaneously injected UV-inactivated SARS coronavirus vaccine elicits systemic humoral immunity in mice. Int. Immunol..

[33] Beck SE, Hull NM, Poepping C, Linden KG (2018). Wavelength-dependent damage to adenoviral proteins across the germicidal UV spectrum. Environ. Sci. Technol..

[34] Araud E, Fuzawa M, Shisler JL, Li J, Nguyen TH (2020). UV inactivation of rotavirus and tulane virus targets different components of the virions. Appl. Environ. Microbiol..

[35] Mackay IM (2004). Real-time PCR in the microbiology laboratory. Clin. Microbiol. Infect..

[36] Rodríguez RA, Pepper IL, Gerba CP (2009). Application of PCR-based methods to assess the infectivity of enteric viruses in environmental samples. Appl. Environ. Microbiol..

[37] Wang J, Mauser A, Chao SF, Remington K, Treckmann R, Kaiser K, Pifat D, Hotta J (2004). Virus inactivation and protein recovery in a novel ultraviolet-C reactor. Vox Sang..

[38] Cutler TD, Zimmerman JJ (2011). Ultraviolet irradiation and the mechanisms underlying its inactivation of infectious agents. Anim. Health Res. Rev..

[39] Trevisan A, Piovesan S, Leonardi A, Bertocco M, Nicolosi P, Pelizzo MG, Angelini A (2006). Unusual high exposure to ultraviolet-C radiation. Photochem. Photobiol..

[40] Narita K, Asano K, Morimoto Y, Igarashi T, Nakane A (2018). Chronic irradiation with 222-nm UVC light induces neither DNA damage nor epidermal lesions in mouse skin, even at high doses. PLoS ONE.

[41] Ratnesar-Shumate S, Williams G, Green B, Krause M, Holland B, Wood S, Bohannon J, Boydston J, Freeburger D, Hooper I (2020). Simulated sunlight rapidly inactivates SARS-CoV-2 on surfaces. J. Infect. Dis..

[42] Hadi J, Dunowska M, Wu S, Brightwell G (2020). Control measures for SARS-CoV-2: A review on light-based inactivation of single-stranded RNA viruses. Pathogens.

[43] De Santis R, Luca V, Faggioni G, Fillo S, Stefanelli P, Rezza G, Lista F (2020). Rapid inactivation of SARS-CoV-2 with LED irradiation of visible spectrum wavelenghts. medRxiv.

[44] Dai T, Gupta A, Murray CK, Vrahas MS, Tegos GP, Hamblin MR (2012). Blue light for infectious diseases: Propionibacterium acnes, Helicobacter pylori, and beyond?. Drug Resist. Updates.

[45] Eickmann M, Gravemann U, Handke W, Tolksdorf F, Reichenberg S, Müller TH, Seltsam A (2018). Inactivation of Ebola virus and Middle East respiratory syndrome coronavirus in platelet concentrates and plasma by ultraviolet C light and methylene blue plus visible light, respectively. Transfusion.

[46] Seghatchian J, Struff WG, Reichenberg S (2011). Main properties of the THERAFLEX MB-plasma system for pathogen reduction. Transfus. Med. Hemother..

[47] Elikaei A, Sharifi Z, Hosseini SM, Latifi H, Musavi Hosseini MK (2014). Inactivation of model viruses suspended in fresh frozen plasma using novel methylene blue based device. Iran. J. Microbiol..

[48] Matsuyama S, Nao N, Shirato K, Kawase M, Saito S, Takayama I, Nagata N, Sekizuka T, Katoh H, Kato F (2020). Enhanced isolation of SARS-CoV-2 by TMPRSS2-expressing cells. Proc. Natl. Acad. Sci. USA.

[49] Reed LJ, Muench H (1938). A simple method of estimating fifty per cent endpoints12. Am. J. Epidemiol..

[50] Shirato K, Nao N, Katano H, Takayama I, Saito S, Kato F, Katoh H, Sakata M, Nakatsu Y, Mori Y (2020). Development of genetic diagnostic methods for detection for novel coronavirus 2019(nCoV-2019) in Japan. Jpn. J. Infect. Dis..

