# Supplementary material for: UVC disinfects SARS-CoV-2 by induction of viral genome damage without apparent effects on viral morphology and proteins
Source: Sci Rep. 2021 Jul 5;11:13804. doi: 10.1038/s41598-021-93231-7 (PMC8257663; doi:10.1038/s41598-021-93231-7)
Supplement: Supplementary file 1 — Supplementary Information. [file 41598_2021_93231_MOESM1_ESM.docx]

**Supplemental Figures**

**Title**

UVC disinfects SARS-CoV-2 by induction of viral genome damage without apparent effects on viral morphology and proteins

**Authors**

Chieh-Wen Lo, Ryosuke Matsuura, Kazuki Iimura, Satoshi Wada, Atsushi Shinjo, Yoshimi Benno, Masaru Nakagawa, Masami Takei and Yoko Aida

**
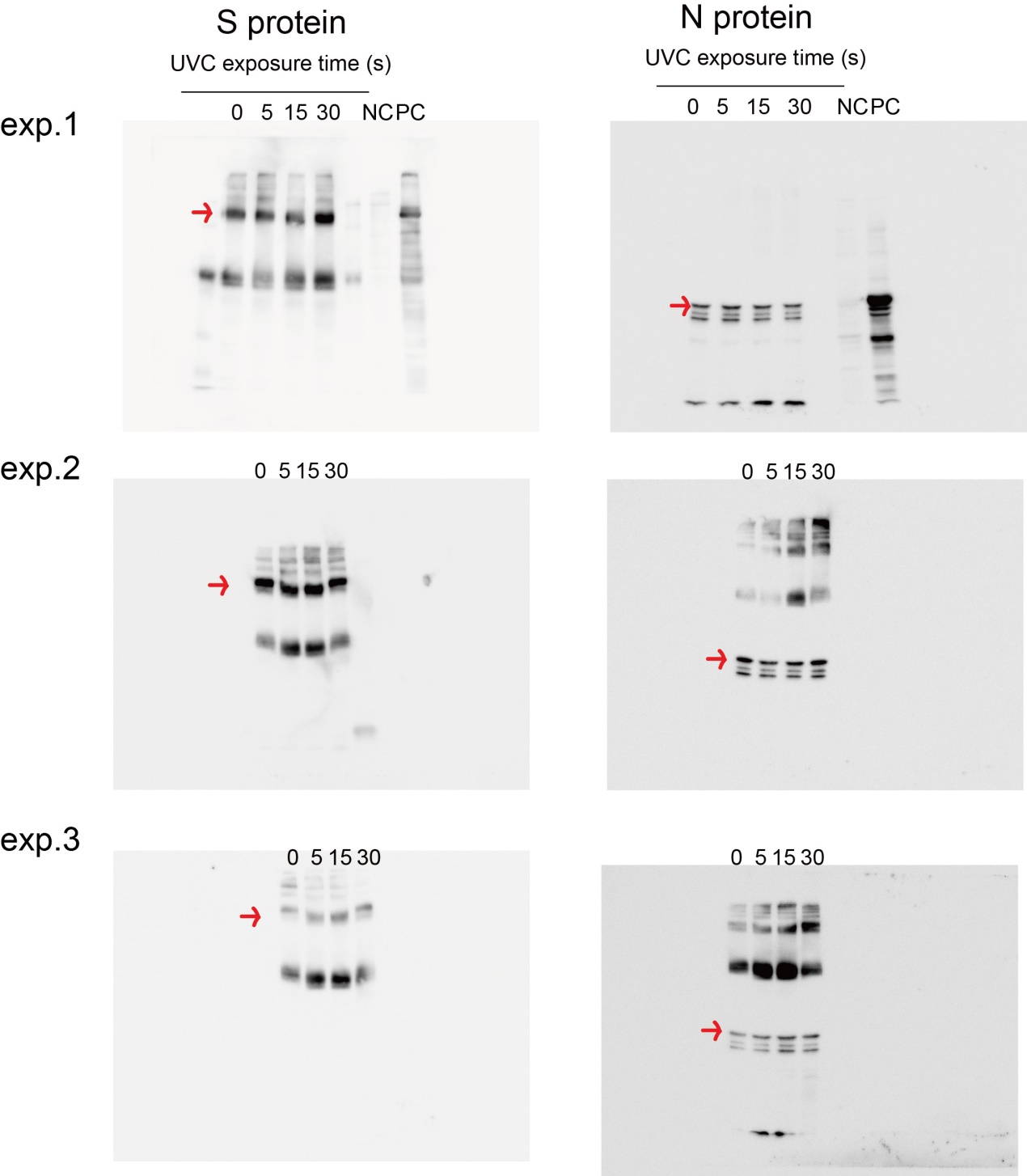
**

**Supplemental Figure S1**. Immunoblot images of UVC irradiated viral spike (S) and nucleocapsid (N) proteins. SARS-CoV-2 (1 mL) with a titer of 1.78 × 10^6^ TCID_50_/mL was irradiated with a UVC light tube for increasing periods of time from 0 to 30 s. Equal volumes of irradiated virus suspension were then used for immunoblotting analysis using anti-SARS-CoV-2 spike monoclonal antibody (1A9) and anti-SARS-CoV-2 nucleocapsid monoclonal antibody (6H3). Positions of the S and N proteins are indicated. NC: negative control. PC: positive control.

**
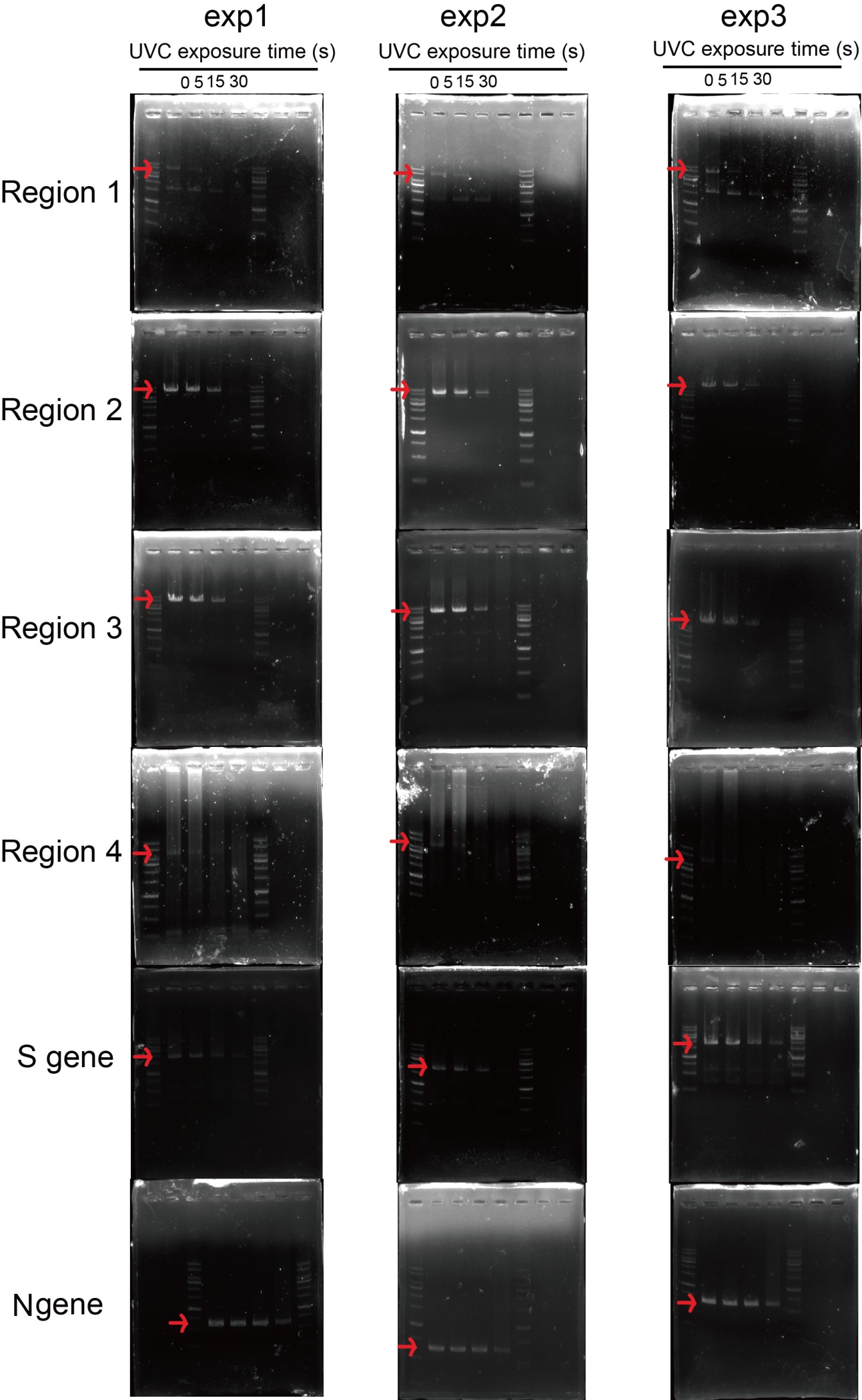
**

**Supplemental Figure S2**. Electrophoresis of long reverse-transcription polymerase chain reaction (RT-PCR) to determine the effect of UVC irradiation on the viral genome. SARS-CoV-2 (5 mL) with a titer of 5 × 10^4^ 50% tissue culture infective dose (TCID_50_/mL) was irradiated with a UVC light tube for increasing periods of time from 0 to 30 s. Viral RNA was reverse transcribed with the reverse primer for each region and then amplified with the corresponding primer sets. The amplified PCR products were used for 0.8% agarose gel electrophoresis and detected by ethidium bromide staining. Positions of the target bands are indicated.
